# Efficacy and immune-inflammatory mechanism of acupuncture-related therapy in animal models of knee osteoarthritis: a preclinical systematic review and network meta-analysis

**DOI:** 10.1186/s13018-024-04660-9

**Published:** 2024-03-08

**Authors:** Yingjie Huang, Hai Huang, Qiqi Chen, Yantong Luo, Jieni Feng, Yuexia Deng, Guangyao Li, Min Li, Jian Sun

**Affiliations:** 1 Present Address: Clinical Medical College of Acupuncture Moxibustion and Rehabilitation, Guangzhou University of Chinese Medicine, Guangzhou, China; 2 Present Address: The Second Clinical College of Guangzhou University of Chinese Medicine, Guangzhou University of Chinese Medicine, Guangzhou, China; 3 The First Clinical College of Guangzhou University of Chinese Medicine, Guangzhou University of Chinese Medicine, Guangzhou, China; 4 Southern Theater General Hospital, Guangzhou, China; 5 Department of traditional Chinese medicine; Guangdong Provincial Key Laboratory of Major Obstetric Diseases; Guangdong Provincial Clinical Research Center for Obstetrics and Gynecology, The Third Affiliated Hospital of Guangzhou Medical University, Guangzhou, China

**Keywords:** Acupuncture, Acupotomy, Knee osteoarthritis, Animal models, Immunity, Inflammation, Systematic review, Network meta-analysis

## Abstract

**Background:**

Many KOA patients have not reached indications for surgery, thus we need to find effective non-surgical treatments. Acupuncture is thought to have the potential to modulate inflammation and cytokines in KOA through the immune system. However, the mechanisms have not been elucidated, and there is no network Meta-analysis of acupuncture on KOA animals. So we evaluate the effect and mechanism of acupuncture-related therapy in KOA animals.

**Methods:**

A comprehensive search was conducted in multiple databases including PubMed, Web of Science, Embase, CBM, CNKI, WanFang, and VIP Database to identify relevant animal studies focusing on acupuncture therapy for KOA. The included studies were assessed for risk of bias using SYRCLE's Risk of Bias tool. Subsequently, pair-wise meta-analysis and network meta-analysis were performed using Stata 15.0 software, evaluating outcomes such as Lequesne index scale, Mankin score, IL-1β, TNF-α, MMP3, and MMP13.

**Results:**

56 RCTs with 2394 animals were included. Meta-analysis showed that among the 6 outcomes, there were significant differences between acupuncture and model group; the overall results of network meta-analysis showed that the normal group or sham operation group performed the best, followed by the acupotomy, acupuncture, and medicine group, and the model group had the worst effect, and there were significant differences between 6 interventions.

**Conclusions:**

Acupuncture-related therapy can be a possible treatment for KOA. The mechanism involves many immune-inflammatory pathways, which may be mediated by DAMPs/TLR/NF-κB/MAPK,PI3K/Akt/NF-κB pathway, or IFN-γ/JAK-STAT pathway. It needs to be further confirmed by more high-quality animal experiments or meta-analysis.

***Systematic review registration*:**

PROSPERO identifier: CRD42023377228.

**Supplementary Information:**

The online version contains supplementary material available at 10.1186/s13018-024-04660-9.

## Introduction

Knee osteoarthritis (KOA) is a prevalent condition among the elderly population worldwide, characterized by the formation of osteophytes and the loss of cartilage in the knee joint. These pathological changes result in restricted knee mobility, stiffness, functional impairment, and a detrimental impact on patients' quality of life [1]. The global incidence of KOA has shown a significant rise, affecting not only older individuals but also younger individuals, highlighting the pressing need to explore more scientific and efficacious treatments for this condition [1, 2]. Presently, there exist limitations in the management of KOA. The therapeutic efficacy of orally administered non-steroidal anti-inflammatory drugs remains suboptimal, and concerns persist regarding their tolerability and safety. While joint replacement and other surgical interventions have proven effective in treating advanced cases, they are accompanied by considerable financial costs, surgical risks, and the potential for implant loosening [3, 4]. Furthermore, a substantial number of KOA patients do not meet the criteria for advanced surgical intervention, underscoring the necessity for the development of effective non-surgical therapies.

Recent authoritative research has demonstrated the remarkable efficacy of acupuncture in treating chronic pain conditions, including KOA, which represents a typical form of chronic pain disease. Clinical investigations have consistently reported that acupuncture effectively alleviates pain intensity, enhances functional mobility, and improves the overall quality of life for patients, leading to its widespread utilization in clinical settings [5, 6]. Moreover, numerous meta-analyses focusing on acupuncture clinical studies involving KOA patients have consistently indicated the potential effectiveness of acupuncture as a therapeutic intervention [7, 8]. Thus, acupuncture is widely acknowledged as an effective and comprehensive treatment modality for KOA.

Although the clinical efficacy of acupuncture in treating KOA has been established, the underlying pathogenesis of KOA and the mechanism of action of acupuncture in treating KOA remain elusive. Furthermore, the assessment of articular cartilage damage in the early stages of clinical KOA is challenging due to a lack of appropriate instruments, necessitating the use of animal models for further study. Animal models have been widely employed in KOA research, with immune-related cells such as macrophage polarization and leukocytes, as well as inflammatory factors like IL-1β and TNF-α, believed to play a crucial role in KOA pathogenesis and progression [9, 10]. Previous animal studies have indicated that acupuncture can decrease the levels of IL-1β, TNF-α, and matrix metalloproteinase-3 (MMP-3) through the NF-κB signaling pathway [17]. However, the precise immune and inflammatory mechanisms involved in acupuncture treatment of KOA animal models remain unclear, and there has been no systematic evaluation of the effects of acupuncture-related therapies on KOA animal models.

Hence, this study represents the inaugural endeavor to conduct a comprehensive network meta-analysis of behavioral testing, tissue staining, cytokines, and immune-inflammatory response pathways associated with acupuncture-related therapies in animal models of KOA. In this study, acupuncture-related therapies to KOA will be evaluated and analyzed, and explored its immune-inflammation mechanism. Provide evidence-based medicine for clinical practice, and to lay the foundation for subsequent research.

## Methods

This study was performed according to the PRISMA 2020 statement [11]. The PRISMA Checklist was provided in the Additional file 1. The study was registered under PROSPERO registration number: CRD42023377228.

### Inclusion and exclusion criteria of literatures

#### Study type

Randomized controlled animal trials.

#### Selection of Studies subjects

Acupuncture-related therapy were used to animals with KOA model.

#### Selection of animals

Study subjects were animals that successfully molded KOA.

#### Intervention measures

Acupuncture-related therapy was used in the test group, which included: manual acupuncture, electroacupuncture, warm acupuncture, acupotomy, and the control group included the normal animals, KOA animals, sham operation, and medicine.

#### Outcome measures

(1) Lequesne index scale; (2) Mankin score; (3) IL-1β; (4) TNF-α; (5) MMP-3; (6) matrix metalloproteinase-13 (MMP-13).

#### Exclusion criteria

(1) studies of non-KOA; (2) non-randomized controlled or quasi-randomized controlled designs of animal experimental; (3) studies of non-acupuncture related; (4) acupuncture with medicine; (5) reviews and meetings; (6) studies with repeated and identical data; (7) lack of original data or incomplete data; (8) non-Chinese or English studies.

### Literature retrieval

Web of science, PubMed, Embase, CBM, CNKI, WanFang and VIP Database were searched by computer. Search terms included: “Acupuncture”, “Manual acupuncture”, “Electroacupuncture”, “acupotomy”, “Warm acupuncture”, “Knee Osteoarthritis”, “Animal Experimentation”. Searches were conducted between the database's establishment and December 2023.

### Literature screening and data extraction

The studies on acupuncture-related therapy for KOA animal models were searched. An independent team of investigators (YJH and HH) independently provided screening, data extraction, and cross-checking. Any disagreements were resolved through discussions with a third party (QQC). Duplicated articles were first excluded at the time of literature screening, then the title was read, after excluding apparently unrelated articles, the abstract was further read. Lastly, the full text was reviewed in order to determine whether they should be included. Data extraction included: (1) basic information included in the study: title, first author; (2) study animal species and interventions; (3) bias risk evaluation; (4) outcome indicators of concern and outcome measurement data; (5) modeling methods and modeling time; (6) acupoints; (7) treatment time and course of treatment each time.

Primary data were not available in the study or were provided in graphical form, we contacted the relevant authors to request the raw data. The data in the graphs were extracted using GetData Graph Digitizer 2.26.

### Risk of bias

The quality assessment risk of bias was conducted using the SYRCLE's Risk of Bias tool [88], which evaluates 10 aspects of the included articles: (1) sequential generation; (2) baseline characteristics; (3) allocation concealment; (4) random housing; (5) blinding; (6) random outcome assessment; (7) blinding; (8) incomplete data reporting; (9) selective outcome reporting; (10) other bias. For each included study, we judged as low bias, high bias and unclear bias(lack of relevant information or uncertain bias). For each included article, the above 10 items were assessed as having a low risk of bias, high risk of bias, or unclear risk of bias. The methodological quality evaluation was performed independently by two reviewers (YJH and HH), and any disagreement was resolved by a third party (QQC).

### Statistics

Pair-wise meta-analysis: we conducted a pair-wise meta-analysis using Stata15.0 software to compare the effects of acupuncture and the Gm on the 6 outcomes. To assess heterogeneity among studies, we calculated I^2^, where I^2^ ≤ 50% indicated little possibility of heterogeneity, while I^2^ > 50% indicated more significant heterogeneity. Meta-analysis with large heterogeneity can find the source of heterogeneity and evaluate the stability of meta results through Subgroup meta-analysis and sensitivity analysis.

Subgroup Meta-analysis: Stata15.0 was used to analyze the Lequesne index scale and Mankin score in subgroups. Different acupuncture methods (manual acupuncture, electroacupuncture, warm acupuncture) in the Ga and treatment courses were grouped to investigate the differences in efficacy between different acupuncture methods and different courses of treatment.

Network meta-analysis: For continuous variables, we reported the standardized mean difference (SMD) and its corresponding 95% confidence interval (CI). Statistical analysis and graphical representation were conducted using Stata15.0 software. The node segmentation model was employed to assess the consistency of direct and indirect comparisons. If the p-value was greater than 0.05, the consistency model was used to indicate agreement between direct and indirect comparisons among interventions. Conversely, if the p-value was less than 0.05, the inconsistency model was employed to identify inconsistencies between direct and indirect comparisons. We calculated the cumulative ranking probabilities for each therapy using surface under the cumulative probability curve area (SUCRA), where a higher SUCRA value indicates a better outcome for the intervention.

## Results

### Literature screening process

**Fig. 1:**
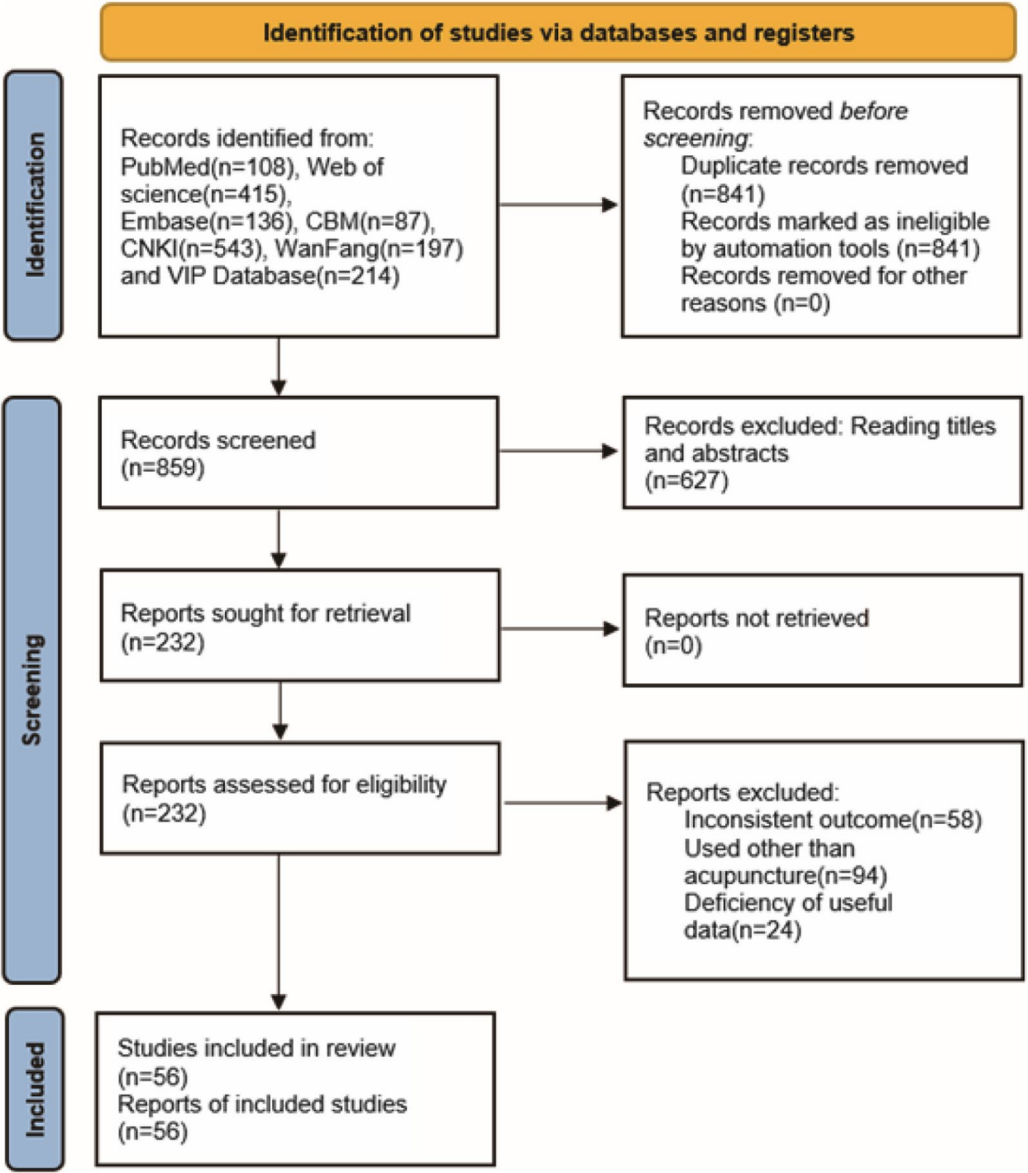
Literature screening process

In accordance with the search strategy set by two independent investigators (YJH and HH), the studies were retrieved from the database. We retrieved a total of 1700 studies and then eliminated 841 duplicates. Screened by reading titles and abstracts, and then removed non-KOA, non-randomized controlled animal studies, non-acupuncture, conference papers, and reviews. Next, by reading the full text, studies that did not involve Lequesne index scale, Mankin score, IL-1β, TNF-α, MMP-3, MMP-13; acupuncture combined with other treatments; and missing data were excluded, and finally 56 articles [12–62, 89–93] were included for quantitative analysis (Fig. 1).

### Literature characteristics

A total of 56 studies were included, with a total of 2394 animals (New Zealand rabbits, SD rats, mice). These included 14 in English and 42 in Chinese. These studies were published from 2006 to 2023.

**Fig. 2:**
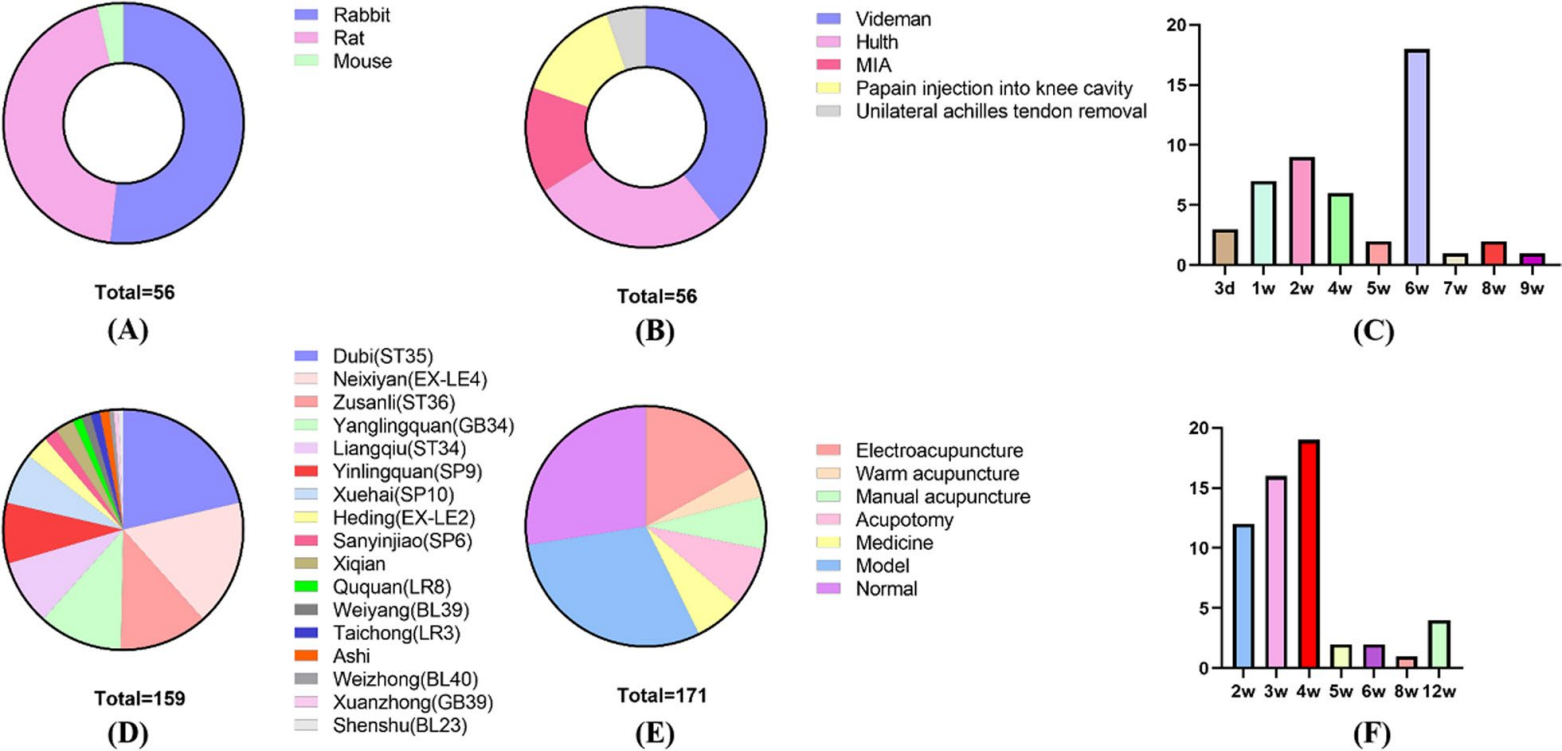
Study characteristics **A** experimental animals, **B** methods of surgical modeling, **C** molding time, d: day, w: week, **D** frequency of acupoint use, **E** interventions, **F** total course of treatment

Model animals: New Zealand rabbits, SD rats, mice (Fig. 2A).

Modeling methods: There were 5 modeling methods, including Videman [13–16, 19, 22, 24, 25, 34, 35, 37–39, 43, 49, 50, 53, 56, 60–62, 92] (22studies), Hulth [12, 17, 18, 20, 21, 23, 31, 33, 36, 40, 42, 44, 52, 90, 91] (15 studies), MIA [28, 30, 41, 46–48, 89, 93] (8 studies), papain injection into the knee joint cavity [26, 32, 45, 54, 55, 57–59] (8 studies), and unilateral hindlimb Achilles tenectomy [27, 29, 51] (3 studies) (Fig. 2B).

Modeling time: Different modeling methods have different modeling time. A total of 49 studies mentioned modeling time. The modeling time of Videman was 5–9 weeks, concentrated in 6 weeks; the modeling time of Hulth was 1–6 weeks, concentrated in 4 weeks and 6 weeks; the modeling time of MIA was 3–2 weeks; the modeling time of papain injection into the knee joint cavity was 1–2 weeks; and the modeling time of unilateral hindlimb Achilles tenectomy was 4 weeks (Fig. 2C).

Frequency of acupoints use: We performed statistics on frequency of acupoints use.The higher frequency of acupoint use was Dubi (ST35) 34 times, Neixiyan (EX-LE4) 27 times, Zusanli (ST36) 19 times, Yanglingquan (GB34) 18 times, Liangqiu (ST34) 15 times, Yinlingquan (SP9) 13 times, Xuehai (SP10) 11 times, Heding (EX-LE2) 5 times, Xiqian 4 times, Sanyinjiao (SP6) 3 times (Fig. 2D).

Intervention measures: including the normal group (Gn), sham operation group (Gso), model group (Gm), acupuncture (Ga), acupotomy (Gao), medicine group (Gmd) (Fig. 2E).

Outcome Measures: Lequesne index scale, Mankin score, IL-1β, TNF-α, MMP-3, MMP-13. 17 studies involved the Lequesne index scale [12, 14, 15, 18, 24, 25, 32, 35–39, 44, 46, 48, 53, 91]; 23 studies involved the Mankin score [13, 14, 17–19, 21, 25, 26, 30, 31, 33–36, 39, 45, 46, 48, 51, 60, 62, 89, 92]; 20 studies mentioned IL-1β [16, 17, 29–32, 36, 41–43, 48, 50, 55–57, 61, 90, 91, 93]; 24 studies mentioned TNF-α [15–17, 28, 29, 36, 40, 42, 43, 46–54, 57, 58, 61, 89, 90, 93]; 13 studies mentioned MMP-3 [15, 17, 19, 22, 27, 36, 42, 44, 52, 53, 58, 59, 91]; 10 studies mentioned MMP-1 [20, 22, 23, 31, 33, 36, 43, 60, 89, 92].

Treatment time and course of treatment: 46 studies mentioned treatment time, 15–30 min, and 10 studies did not clearly mention the treatment time. 55 studies mentioned the intervention course, which ranged from 2 to 12 weeks, and 1 study did not explicitly mention the course (Fig. 2F).

### Publication bias

**Fig. 3:**
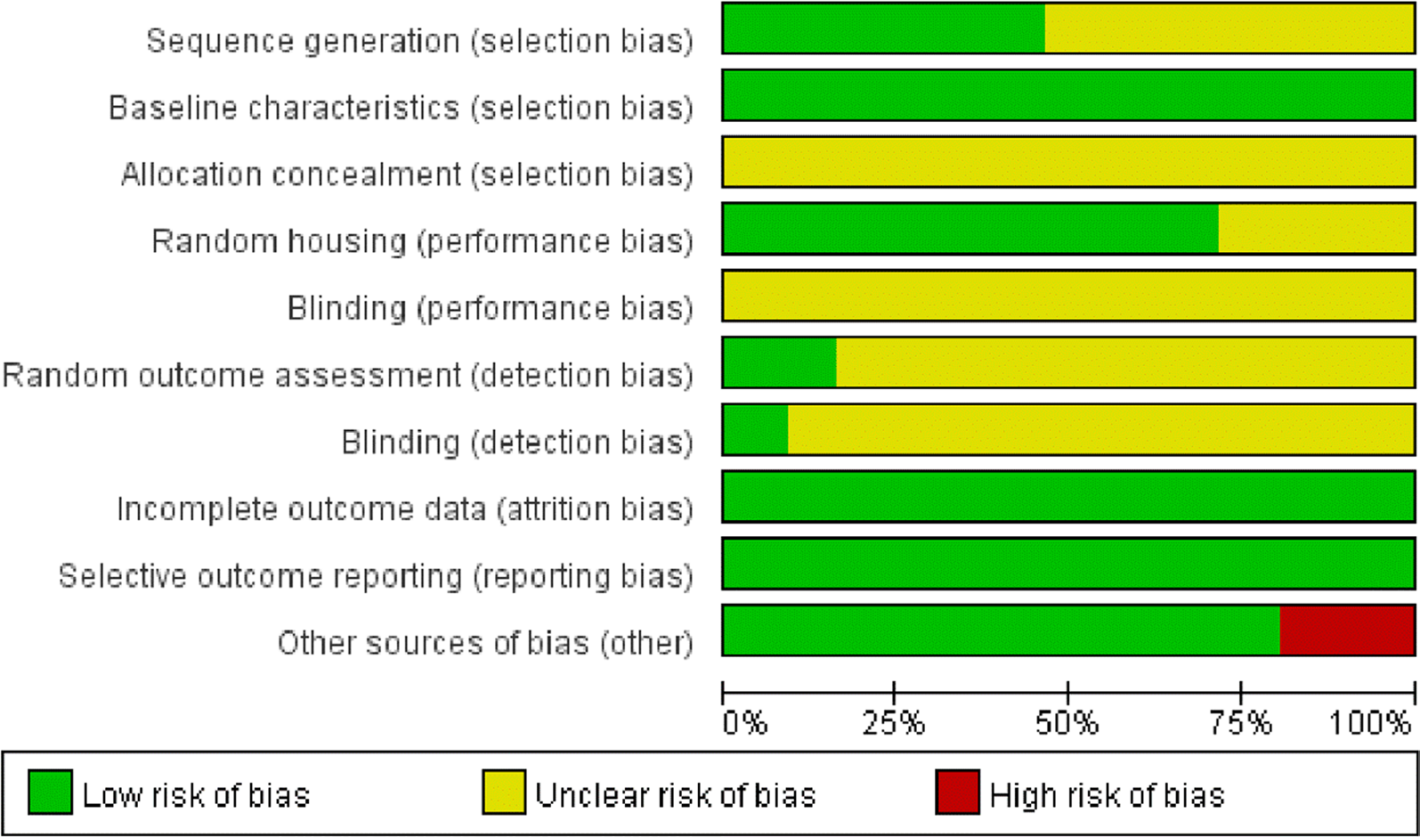
Results of the risk of bias evaluation

Overall, included studies were of moderate quality. The evaluation results are as follows: all studies were randomized, with 26 of them explicitly mentioning the animal randomization method [12, 14, 15, 17, 18, 21, 22, 25–30, 32, 33, 42–44, 46, 48, 52, 61, 62, 90–92], all of which utilized random number methods. However, the remaining 30 articles did not provide specific details regarding the mode of randomization.The baseline characteristics of the animals included in the studies did not show statistically significant differences, but none mentioned allocation concealment. None of the articles explicitly mentioned that animals were randomly assigned to housing, but 40 studies [12–15, 17, 19–22, 24–26, 28, 30–41, 43, 44, 46–49, 54, 55, 57, 59, 89–93] described animal housing conditions, and the remaining 16 studies lacked explicit description of the housing conditions for the animals.Given that the interventions in these studies involved acupuncture or acupotomy, blinding of the investigators was not feasible. Out of the 56 studies evaluated for results, 10 studies [30, 37–41, 47, 48, 54, 61] randomly selected animals for outcome assessment, while the remaining 45 studies did not clearly specify whether animals were randomly selected for evaluation. In terms of blinding implementation, five studies. 5 studies [25, 33, 34, 40, 41] explicitly mentioned blinding in the outcome statistics, whereas the remaining studies did not provide explicit information regarding blinding. It is worth noting that all included literature data were complete without any selective reporting of results.Regarding other sources of bias, 10 studies [14, 21, 28, 37–39, 41, 48, 49, 53] did not explicitly mention the timing of each treatment session, and 1 study [40] did not explicitly mention the course of treatment (Fig. 3 and Additional file 2: Fig. 1).

### Pair-wise meta-analysis

In order to clearly show the efficacy of acupuncture in the treatment of KOA, we did a meta-analysis of Ga compared with the Gm. The meta-analysis results showed that in the Lequesne index scale, a comparison was made between Ga and Gm (SMD: − 3.02; 95% CI − 3.94, − 2.09, *P* < 0.05); in the Mankin score, a comparison was made between the acupuncture and Gm (SMD: − 3.57; 95% CI − 4.38, − 2.77, *P* < 0.05); in IL-1β, a comparison was made between Ga and Gm (SMD: − 3.48; 95% CI − 4.28, − 2.68, *P* < 0.05); in TNF-α, a comparison was made between Ga and Gm (SMD: − 3.27; 95% CI − 4.16, − 2.39, *P* < 0.05); in MMP-3, a comparison was made between Ga and Gm (SMD: − 3.68; 95% CI − 4.81, − 2.54, *P* < 0.05); in MMP-13, a comparison was made between Ga and Gm (SMD: − 4.06; 95% CI − 5.22, − 2.89, *P* < 0.05), the differences were statistically significant(*P* < 0.05). These results indicated that among these 6 outcomes, acupuncture was superior to the model animals (Additional file 2: Fig. 2).

### Subgroup meta-analysis

Lequesne index scale is an important behavioral evaluation of KOA, Mankin score is the score of HE staining and SO staining results of KOA articular cartilage, and pathological staging is performed according to it. These outcomes are important for the determination of efficacy. Therefore, we performed subgroup meta-analysis of Lequesne index scale and Mankin score according to different acupuncture methods and different courses of treatment.

**Fig. 4:**
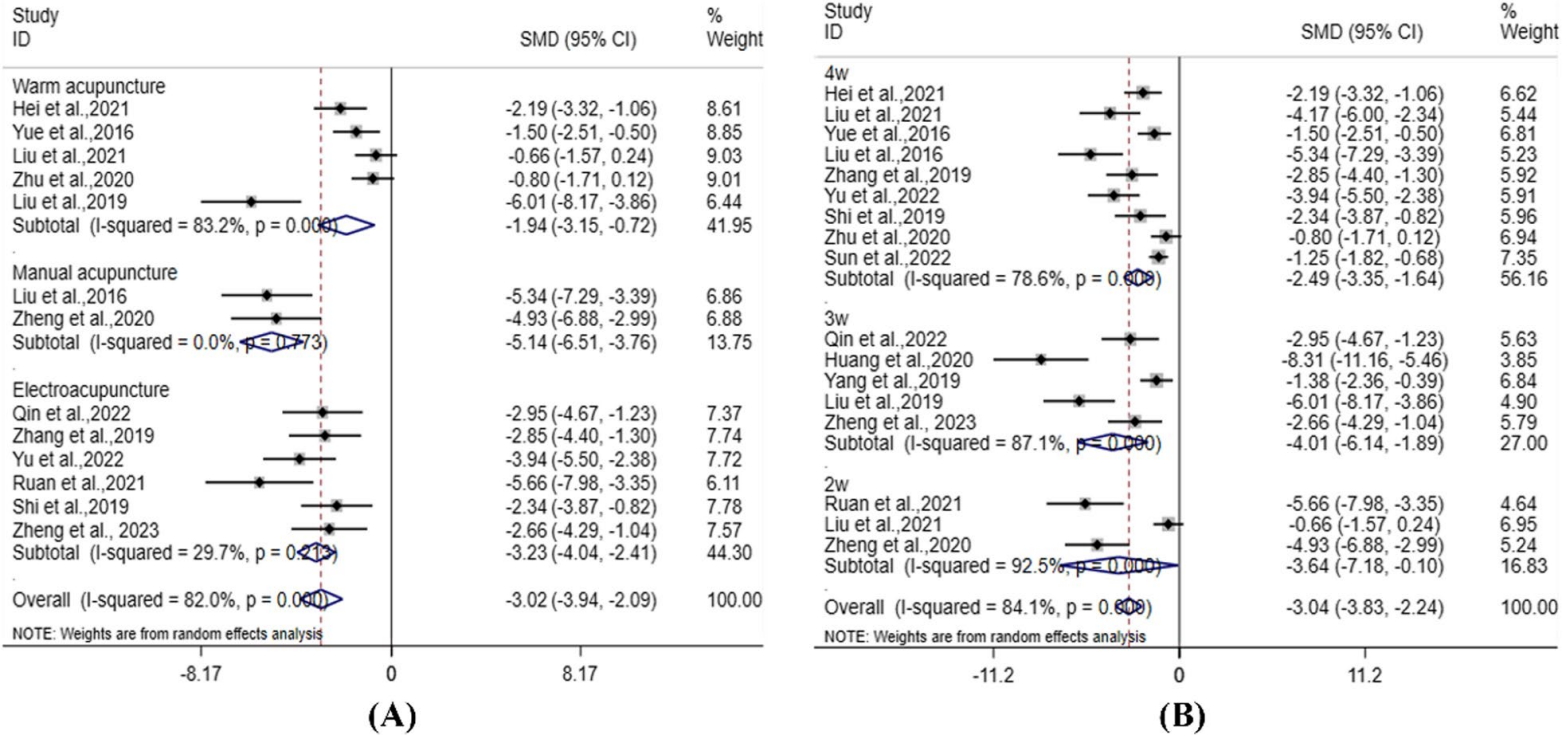
Subgroup Meta-analysis of Lequesne index scale. **A** Acupuncture methods; **B** Courses of treatment

Lequesne index scale: 13 studies [12, 14, 18, 24, 25, 32, 35, 36, 39, 44, 46, 53, 91] mentioned different acupuncture methods, which were divided into manual acupuncture, electroacupuncture, and warm acupuncture (Fig. 4A). Compared with Gm, the combined value of acupuncture (SMD: − 3.02; 95% CI − 3.94, − 2.41; I^2^ = 82.0%) was statistically significant, *P* < 0.05. Among them, warm acupuncture (SMD: − 1.94; 95% CI − 3.15, − 0.72; I^2^ = 83.2%); manual acupuncture (SMD: − 5.14; 95% CI − 6.51, − 3.76; I^2^ = 0.0%); and electroacupuncture (SMD: − 3.23; 95% CI − 4.04, − 2.41; I^2^ = 29.7%) were statistically significant, *P* < 0.05. These results indicated that different acupuncture types in Ga were better than those in Gm. 17 studies [12, 14, 15, 18, 24, 25, 32, 35–39, 44, 46, 48, 53, 91] mentioned different courses of treatment, divided into 2w, 3w, 4w (Fig. 4B). Compared with the Gm, the Ga and Gao (SMD: − 3.04; 95% CI − 3.91, − 2.24; I^2^ = 84.1%) was statistically significant, *P* < 0.05. Among them, 2w (SMD: − 3.64; 95% CI − 7.18, − 0.10; I^2^ = 92.5%), 3w (SMD: − 4.01; 95% CI − 6.14, − 1.89; I^2^ = 87.1%), and 4w (SMD: − 2.49; 95% CI − 3.35, − 1.64; I^2^ = 78.6%) were statistically significant, *P* < 0.05. It showed that acupuncture and acupotomy were superior to the Gm in different courses of treatment.

**Fig. 5:**
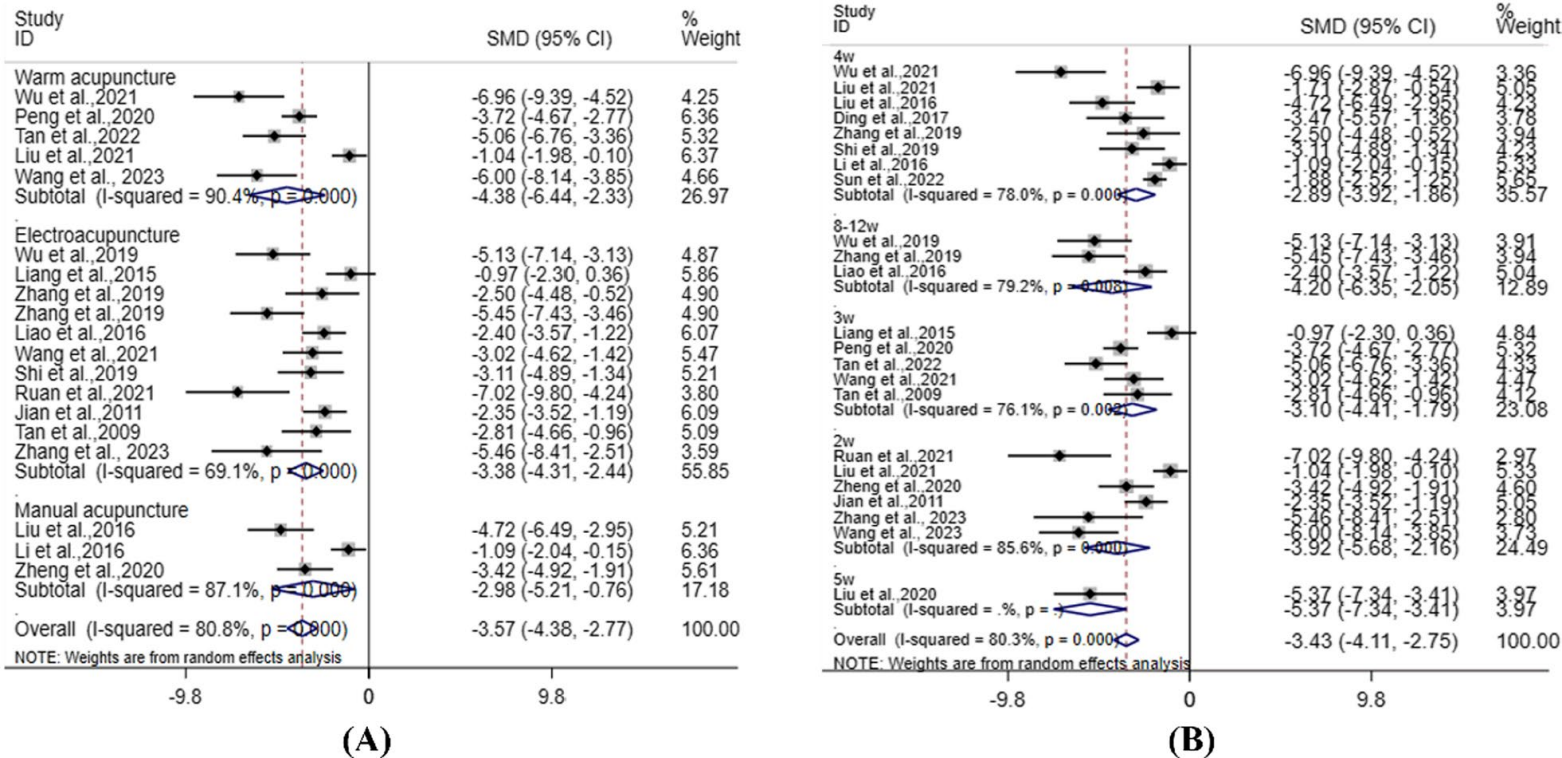
Subgroup Meta-analysis of Mankin. **A** Acupuncture methods; **B** Courses of treatment

Mankin score: 19 studies [13, 17–19, 25, 26, 30, 31, 33–36, 39, 45, 46, 60, 62, 89, 92] mentioned different acupuncture methods, which were divided into manual acupuncture, electroacupuncture, and warm acupuncture (Fig. 5A). Compared with the Gm, the combined value of acupuncture (SMD: − 3.57; 95% CI − 4.38, − 2.77; I^2^ = 80.8%) was statistically significant, *P* < 0.05. Among them, warm acupuncture (SMD: − 4.38; 95% CI − 6.44, − 2.33; I^2^ = 90.4%); manual acupuncture (SMD: − 2.98; 95% CI − 5.21, − 0.76; I^2^ = 87.1%); and electroacupuncture (SMD: − 3.38; 95% CI − 4.31, − 2.44; I^2^ = 69.1%) were statistically significant, *P* < 0.05. These results indicated that different acupuncture types in the Ga were better than those in the Gm. 23 studies [13, 14, 17–19, 21, 25, 26, 30, 31, 33–36, 39, 45, 46, 48, 51, 60, 62, 89, 92] mentioned different courses of treatment, divided into 2w, 3w, 4w, 5w, 8-12w (Fig. 5B). Compared with the Gm, the combined value of Ga and Gao combination (SMD:-3.43; 95% CI − 4.11, − 2.75; I^2^ = 80.3%) was statistically significant, *P* < 0.05. Among them, 2w (SMD: − 3.92; 95% CI − 5.68, − 2.16; I^2^ = 85.6%), 3w (SMD: − 3.10; 95% CI − 4.41, − 1.79; I^2^ = 76.1%), 4w (SMD: − 2.89; 95% CI − 3.92, − 1.86; I^2^ = 78.0%), 5w (SMD: − 5.37; 95% CI − 7.34, − 3.41), 8-12w (SMD: − 4.20; 95% CI − 6.35, − 2.05; I^2^ = 79.2%) were statistically significant, *P* < 0.05. It showed that acupuncture and acupotomy were superior to the Gm in different courses of treatment.

### Heterogeneity analysis

There are heterogeneous results in meta-analysis. Analyzing the raw data revealed that the included studies might have methodological heterogeneity due to less description of blinding and concealment of allocation. Furthermore, clinical heterogeneity may result from acupoints, surgical modeling methods, modeling time and other factors. However, in the original study, these details were not specifically described and some of the results came from a small number of studies, it was not possible to further investigate the source of heterogeneity by subgroup meta-analysis. We performed subgroup meta-analysis of two important indicators, acupuncture methods, treatment courses of Lequesne index scale and Mankin score, in which different types of acupuncture method in Lequesne index scale may be the source of heterogeneity, and the remaining subgroup analysis did not eliminate heterogeneity. However, we did a sensitivity analysis (Additional file 2: Fig. 3) and found that after excluding any of the studies, the Meta-analysis results remained stable. Therefore, this heterogeneity seems inevitable, and the random effects model was used for the meta-analysis.

### Network meta-analysis

**Fig. 6:**
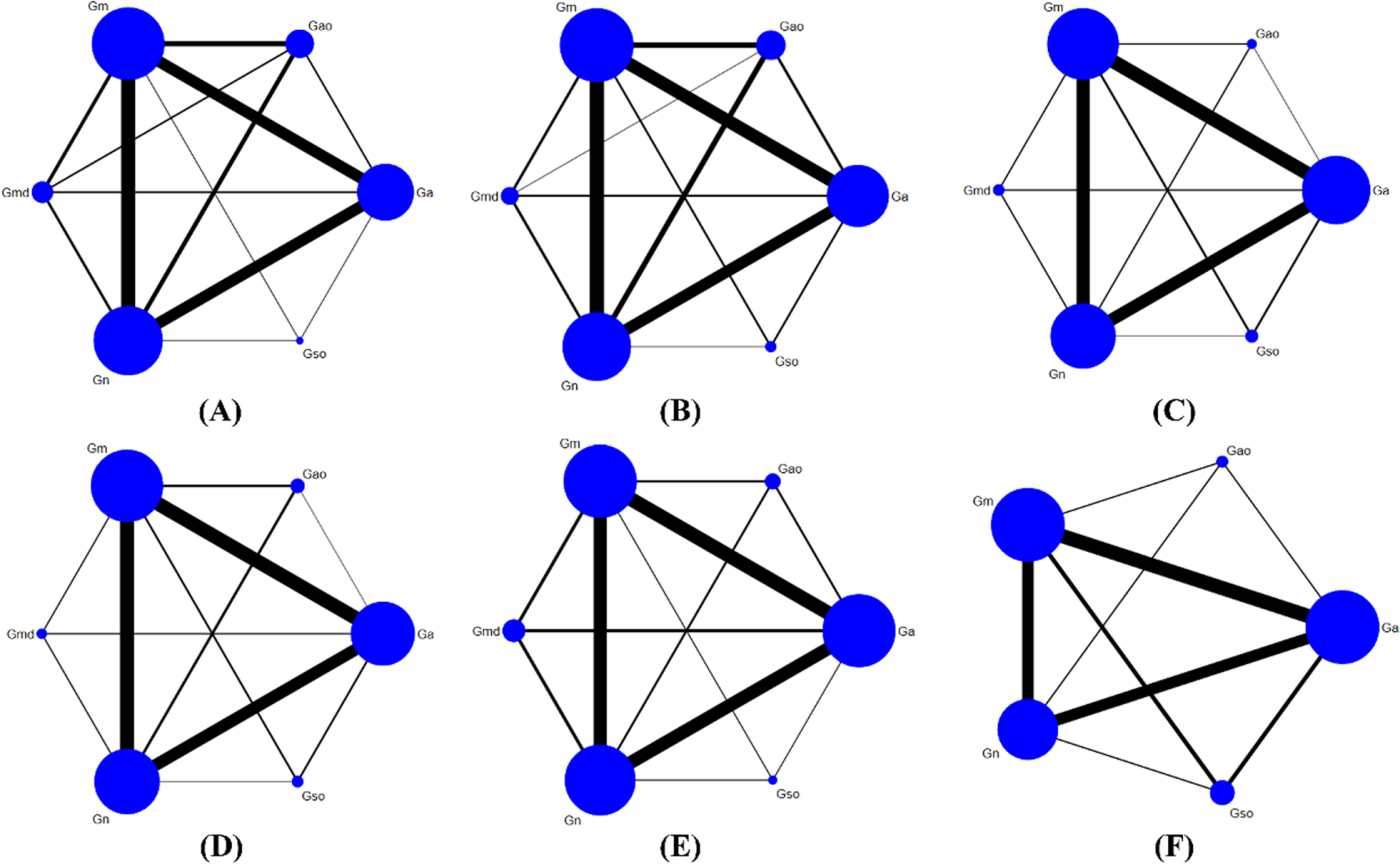
Evidence network diagram **A** Lequesne index scale, **B** Mankin score, **C** IL-1β, **D** TNF-α, **E** MMP-3, **F** MMP-13

Network Meta-analysis was performed on the 6 outcomes (Fig. 6). A node splitting model was utilized to assess the agreement between direct and indirect comparisons for the six outcome measures tested. The analysis showed that the p-values for the node splitting model were greater than 0.05, suggesting no significant disagreement between direct and indirect comparisons. Similarly, the consistency model results also indicated agreement between direct and indirect comparisons, with p > 0.05.

#### Lequesne index scale

Lequesne index scale is an important behavioral evaluation of KOA. A total of 17 studies [12, 14, 15, 18, 24, 25, 32, 35–39, 44, 46, 48, 53, 91] involved the Lequesne index scale in 551 animals, with a total of 6 interventions (Fig. 6A).

Network Meta-analysis results showed that compared with the Gm, the Gn, Ga, Gmd, and Gao were statistically significant (*P* < 0.05), indicating that the Gn, Ga, Gmd, and Gao were superior to the Gm; the Gn was statistically significant with the other 5 interventions (*P* < 0.05), indicating that the Gn was superior to other interventions. However, there was no statistical significance among Ga, Gao, Gmd, and Gso (P > 0.05) (Table 1A).

**Table 1:** Network meta-analysis

		SMD (95% CI)			
*(A)*					
Gn	3.05 (1.52, 4.59)	3.24 (1.43, 5.05)	3.39 (2.27, 4.51)	5.87 (2.44, 9.31)	6.59 (5.43, 7.75)
− **3.05 (**− **4.59, **− **1.52)**	Gao	0.19 (− 1.81, 2.19)	0.34 (− 1.28, 1.96)	2.82 (− 0.81, 6.45)	3.54 (2.04, 5.04)
− **3.24 (**− **5.05, **− **1.43)**	− 0.19 (− 2.19, 1.81)	Gmd	0.15 (− 1.70, 2.00)	2.63 (− 1.11, 6.38)	3.35 (1.58, 5.12)
− **3.39 (**− **4.51, **− **2.27)**	− 0.34 (− 1.96, 1.28)	− 0.15 (− 2.00, 1.70)	Ga	2.48 (− 0.93, 5.89)	3.20 (2.09, 4.31)
− **5.87 (**− **9.31, **− **2.44)**	− 2.82 (− 6.45, 0.81)	− 2.63 (− 6.38, 1.11)	− 2.48 (− 5.89, 0.93)	Gso	0.72 (− 2.67, 4.10)
− **6.59 (**− **7.75, **− **5.43)**	− **3.54 (**− **5.04, **− **2.04)**	− **3.35 (**− **5.12, **− **1.58)**	− **3.20 (**− **4.31, **− **2.09)**	− 0.72 (− 4.10, 2.67)	Gm
*(B)*					
Gso	1.14 (− 1.21, 3.50)	4.63 (2.03, 7.22)	4.78 (2.45, 7.10)	5.54 (2.71, 8.38)	8.44 (6.03, 10.86)
− 1.14 (− 3.50, 1.21)	Gn	3.48 (2.18, 4.79)	3.63 (2.66, 4.61)	4.40 (2.64, 6.15)	7.30 (6.27, 8.33)
− **4.63 (**− **7.22, **− **2.03)**	− **3.48 (**− **4.79, **− **2.18)**	Gao	0.15 (− 1.22, 1.51)	0.92 (− 1.06, 2.90)	3.81 (2.50, 5.13)
− **4.78 (**− **7.10, **− **2.45)**	− **3.63 (**− **4.61, **− **2.66)**	− 0.15 (− 1.51, 1.22)	Ga	0.77 (− 0.98, 2.52)	3.67 (2.73, 4.60)
− **5.54 (**− **8.38, **− **2.71)**	− **4.40 (**− **6.15, **− **2.64)**	− 0.92 (− 2.90, 1.06)	− 0.77 (− 2.52, 0.98)	Gmd	2.90 (1.17, 4.63)
− **8.44 (**− **10.86, **− **6.03)**	− **7.30 (**− **8.33, **− **6.27)**	− **3.81 (**− **5.13, − 2.50)**	**− 3.67 (− 4.60, − 2.73)**	**− 2.90 (− 4.63, − 1.17)**	Gm
*(C)*					
Gn	0.39 (− 1.60, 2.37)	1.28 (− 0.86, 3.43)	1.86 (0.98, 2.74)	3.23 (1.04, 5.43)	5.71 (4.60, 6.82)
− 0.39 (− 2.37, 1.60)	Gso	0.90 (− 1.89, 3.68)	1.48 (− 0.42, 3.37)	2.85 (0.01, 5.68)	5.22 (3.28, 7.16)
− 1.28 (− 3.43, 0.86)	− 0.90 (− 3.68, 1.89)	Gmd	0.58 (− 1.55, 2.71)	1.95 (− 1.03, 4.92)	4.32 (2.15, 6.49)
**− 1.86 (− 2.74, − 0.98)**	− 1.48 (− 3.37, 0.42)	− 0.58 (− 2.71, 1.55)	Ga	1.37 (− 0.84, 3.58)	3.74 (− 2.81, 4.67)
**− 3.23 (− 5.43, − 1.04)**	**− 2.85 (− 5.68, − 0.01)**	− 1.95 (− 4.92, 1.03)	− 1.37 (− 3.58, 0.84)	Gao	2.37 (− 0.18, 4.57)
**− 5.61 (− 6.66, − 4.56)**	**− 5.22 (− 7.16, − 3.28)**	**− 4.32 (− 6.49, − 2.15)**	− 3.74 (− 4.67, 2.81)	− 2.37 (− 4.57, 0.18)	Gm
*(D)*					
Gn	0.67 (− 3.49, 4.83)	1.95 (− 1.52, 5.42)	2.38 (0.57, 4.19)	2.89 (− 1.76, 7.55)	6.89 (4.93, 8.86)
− 0.67 (− 4.83, 3.49)	Gso	1.28 (− 3.93, 6.50)	1.72 (− 2.28, 5.71)	2.23 (− 3.75, 8.21)	6.23 (2.20, 10.25)
− 1.95 (− 5.42, 1.52)	− 1.28 (− 6.50, 3.93)	Gao	0.43 (− 3.22, 4.08)	0.94 (− 4.72, 6.60)	4.94 (1.35, 8.53)
**− 2.38 (− 4.19, − 0.57)**	− 1.72 (− 5.71, 2.28)	− 0.43 (− 4.08, 3.22)	Ga	0.51 (− 4.12, 5.15)	4.51 (2.69, 6.33)
− 2.89 (− 7.55, 1.76)	− 2.23 (− 8.21, 3.75)	− 0.94 (− 6.60, 4.72)	− 0.51 (− 5.15, 4.12)	Gmd	4.00 (− 0.66, 8.66)
**− 6.89 (− 8.86, − 4.93)**	**− 6.23 (− 10.25, − 2.20)**	**− 4.94 (− 8.53, − 1.35)**	**− 4.51 (− 6.33, − 2.69)**	− 4.00 (− 8.66, 0.66)	Gm
*(E)*					
Gn	0.37 (− 5.30, 6.03)	2.04 (− 2.05, 6.12)	2.81 (0.84, 4.78)	3.13 (− 0.25, 6.50)	6.57 (4.42, 8.71)
− 0.37 (− 6.03, 5.30)	Gso	1.67 (− 5.11, 8.45)	2.44 (− 3.20, 8.09)	2.76 (− 3.62, 9.14)	6.20 (0.53, 11.87)
− 2.04 (− 6.12, 2.05)	− 1.67 (− 8.45, 5.11)	Gao	0.78 (− 3.28, 4.83)	1.09 (− 3.94, 6.12)	4.53 (0.45, 8.62)
**− 2.81 (− 4.78, − 0.84)**	− 2.44 (− 8.09, 3.20)	− 0.78 (− 4.83, 3.28)	Ga	0.31 (− 3.04, 3.67)	3.76 (1.82, 5.70)
− 3.13 (− 6.50, 0.25)	− 2.76 (− 9.14, 3.62)	− 1.09 (− 6.12, 3.94)	− 0.31 (− 3.67, 3.04)	Gmd	3.44 (0.03, 6.86)
**− 6.57 (− 8.71, − 4.42)**	**− 6.20 (− 11.87, − 0.53)**	**− 4.53 (− 8.62, − 0.45)**	**− 3.76 (− 5.70, − 1.82)**	**− 3.44 (− 6.86, − 0.03)**	Gm
*(F)*					
Gn	2.15 (− 1.46, 5.77)	3.27 (1.01, 5.53)	4.22 (− 1.11, 9.54)	7.88 (5.48, 10.27)	
− 2.15 (− 5.77, 1.46)	Gso	1.11 (− 2.17, 4.40)	2.06 (− 3.97, 8.10)	5.72 (2.38, 9.06)	
**− 3.27 (− 5.53, − 1.01)**	− 1.11 (− 4.40, 2.17)	Ga	0.95 (− 4.33, 6.23)	4.61 (2.55, 6.66)	
− 4.22 (− 9.54, 1.11)	− 2.06 (− 8.10, 3.97)	− 0.95 (− 6.23, 4.33)	Gao	3.66 (− 1.64, 8.96)	
**− 7.88 (− 10.27, − 5.48)**	**− 5.72 (− 9.06, − 2.38)**	**− 4.61 (− 6.66, − 2.55)**	− 3.66 (− 8.96, 1.64)	Gm	

(A) Lequesne index scale, (B) Mankin score, (C) IL-1β, (D) TNF-α, (E) MMP-3, (F) MMP-13

Bolded font indicates that there was a significant difference between these two interventions (*P* < 0.05)

SUCRA results showed that Gn (100.0%) > Gao (63.3%) > Gmd (58.4%) > Ga (53.9%) > Gso (17.7%) > Gm (6.8%) (Additional file 2: Fig. 4(A)).

#### Mankin score

Mankin score is the score of HE staining and SO staining results of KOA articular cartilage, and pathological staging is performed accordingly.There were 23 studies [13, 14, 17–19, 21, 25, 26, 30, 31, 33–36, 39, 45, 46, 48, 51, 60, 62, 89, 92], 735 animals involved Mankin score, and there were 6 interventions (Fig. 6B).

Network Meta-analysis showed that compared with the Gm, the Gn, Ga, Gmd, Gao and Gso had statistical significance (*P* < 0.05), indicating that these 5 interventions were superior to the Gm; compared with the Ga, Gmd, Gao and Gm, the Gn and Gso had statistical significance (*P* < 0.05), indicating that the Gn and Gso were superior to the Ga, Gmd, Gao and Gm. However, there was no statistical significance among Ga, Gmd, and Gao (*P* > 0.05) (Table 1B).

SUCRA results showed that Gso (96.7%) > Gn (83.3%) > Gao (48.0%) > Ga (44.3%) > Gmd (27.7%) > Gm (0.0%) (Additional file 2: Fig. 4B).

#### 3.7.3 IL-1β

IL-1β can be secreted by immune macrophages and helper T cells and is an important inflammatory indicator of KOA, which mediates the inflammatory response and destroys cartilage. 20 studies [16, 17, 29–32, 36, 41–43, 48, 50, 55–57, 61, 89–91, 93] involved IL-1β in 656 animals, with a total of 6 interventions (Fig. 6C).

Network Meta-analysis showed that compared with the Gm, the Gn, Ga, Gmd, Gao and Gso were statistically significant (*P* < 0.05), indicating that these 5 interventions were superior to the Gm; the Gn was statistically significant compared with the Ga, Gao and Gm (*P* < 0.05), indicating that the Gn was superior to the Ga, Gao and Gm. The Gso was statistically significant compared with the Gao (*P* < 0.05). There was no statistical significance among Ga, Gmd and Gso (P > 0.05) (Table 1C).

SUCRA results showed that Gn (90.6%) > Gso (79.6%) > Gmd (60.2%) > Ga (45.0%) > Gao (24.2%) > Gm (0.4%) (Additional file 2: Fig. 4C).

#### TNF-α

TNF-α is an important inflammatory marker in KOA and is secreted by macrophages and helper T cells. 24 studies [15–17, 28, 29, 36, 40, 42, 43, 46–54, 57, 58, 61, 89, 90, 93] involved TNF-α in 910 animals, with a total of 6 interventions (Fig. 6D).

Network Meta-analysis showed that compared with the Gm, the Gn, Ga, Gao and Gso were statistically significant (*P* < 0.05), indicating that these 4 interventions were superior to the Gm. The Gn was statistically significant compared with the Ga (*P* < 0.05). There was no significant difference among Gmd, Gao, Gso and Gn (P > 0.05) (Table 1D).

SUCRA results showed that Gn (87.5%) > Gso (72.0%) > Gao (53.3%) > Ga (44.0%) > Gmd (42.2%) > Gm (1.0%) (Additional file 2: Fig. 4D).

#### MMP-3

MMP-3 is stimulated by the inflammatory factors such as IL-1β, TNF-α, and EGF, and it can degrade the collagen in the cartilage. While MMP-3 is abnormally elevated in a variety of immune diseases. There were 13 studies [15, 17, 19, 22, 27, 36, 42, 44, 52, 53, 58, 59, 91], 469 animals involved MMP-3, and there were a total of 6 interventions (Fig. 6E).

Network Meta-analysis showed that compared with the Gm, the Gn, Ga, Gao, Gmd and Gso were statistically significant (*P* < 0.05), indicating that these 5 interventions were superior to the Gm; the Gn was statistically significant compared with the Ga (*P* < 0.05), indicating that the Gn was superior to Ga. There was no statistical significance among Gao, Gao, Gmd and Gso (P > 0.05) (Table 1E).

SUCRA results showed that Gn (86.9%) > Gso (74.4%) > Gao (55.5%) > Ga (42.5%) > Gmd (39.4%) > Gm (1.2%) (Additional file 2: Fig. 4E).

#### MMP-13

MMP-13 degrades type II collagen, destroys cartilage structure, and further provokes inflammatory and immune responses. There were 10 studies [20, 22, 23, 31, 33, 36, 43, 60, 89, 92], 285 animals involved MMP-13, and there were a total of 5 interventions (Fig. 6F).

Network Meta-analysis showed that compared with the Gm, the Gn, Ga, and Gso were statistically significant (*P* < 0.05), indicating that these 3 interventions were superior to the Gm; the Gn was statistically significant compared with the Ga (*P* < 0.05), indicating that the Gn was superior to the Ga. There was no statistical significance among Ga, Gao and Gso (P > 0.05). There was no statistical significance between the Gn and the sham group (P > 0.05) (Table 1F).

SUCRA results showed that Gn (95.5%) > Gso (65.3%) > Ga (47.2%) > Gao (39.7%) > Gm (2.2%) (Additional file 2: Fig. 4F).

## Discussion

In recent years, more and more clinical trials and animal experiments have showcased the effectiveness of acupuncture in treating KOA [63, 64], due to the lack of ideal results of existing therapeutic measures, while acupuncture-related therapy plays an important role in treating KOA because it is a safe and reliable alternative therapy. However, the mechanism of acupuncture-related therapy on immune-inflammation in treating KOA lacks systematic analysis. In light of this, a network meta-analysis was conducted for the first time to comprehensively assess the efficacy and underlying mechanisms of acupuncture-related therapies in animal models of KOA.

## Summary of results

This study included 56 studies. The findings revealed significant differences between the Ga and the Gm across the six outcome measures; Subgroup meta-analysis results indicated that different acupuncture methods in the Ga were superior to those in the Gm in the Lequesne index scale and Mankin score. Meanwhile different courses of acupuncture and acupotomy were more effective than those in the Gm, indicating that acupuncture treatment for KOA can shorten the course of treatment, and has more prominent advantages in improving the inflammatory response and joint function, reducing the patient 's symptoms. Network meta-analysis showed that the Gn or Gso showed the best performance, followed by the Gao, Ga, and Gmd, and the Gm had the worst effect, and there were significant differences between the Gn, Gso, Gao, Ga, and Gmd and the Gm, and there was no significant difference between the Gao, Ga, and Gmd. Thus, in addition to the Gn and Gso, acupuncture and acupotomy treatment of KOA animal models had a better effect.

### Possible mechanisms

Modern medicine believes that the pathology of KOA is characterized by degeneration of articular cartilage, formation of bone redundancy, synovitis, and degeneration of connective tissues such as ligaments and menisci [65, 94]. Synovitis is considered a common finding in KOA [65]. The development of synovitis involves immune response, primarily associated with synovial macrophage polarization and T cells. Synovial macrophages are activated to produce cytokines such as IL-1β TNF-α, which destroy cartilage. Acupuncture can effectively stimulate immune cytokines to repair connective tissue damage, and can affect synovial macrophage M1/M2 polarization by regulating T helper cells, which can play an anti-inflammatory and analgesic role [65]. Therefore, this study investigated how acupuncture modulates the activity of immune cells and inflammatory factors to treat KOA.

### Role and activation of macrophages in KOA immunity

According to studies, synovial macrophages have the crucial role in the inflammation of KOA synovium and aggravate inflammation of KOA synovium [66]. Synovial macrophage activation correlates with KOA severity and symptoms [67]. Macrophages are key contributors to both the innate and adaptive immunity involved in KOA. The innate immune system comprises various cell types, including macrophages, neutrophils, and natural killer cells. Activation of the innate immune system occurs when surface expression pattern recognition receptors bind to pathogen-associated molecular patterns (PAMPs) and danger-associated molecular patterns (DAMPs) [68]. At the same time, T helper cells of adaptive immunity can affect the polarization of macrophages through secreted cytokines, so that macrophages play a role through innate and adaptive immunity [69]. Macrophages can polarize into M1 and M2 types through multiple signaling pathways such as PI3K/Akt, IFN-γ/JAK-STAT, and TLR/NF-κB, of which M1 macrophages can release cytokines that are pro-inflammatory [70, 95]. M1 macrophages can signal and activate inflammatory mediators interferon-γ (IFNγ), lipopolysaccharide (LPS), TNF-α or PAMPs, thereby releasing pro-inflammatory factors, and activate lymphocytes or other immune cells to kill pathogens and trigger inflammation [71].

### Macrophages mediate IL-1β, TNF-α

The results showed that Ga and Gao significantly decreased IL-1β and TNF-α in KOA animal models. It is worth exploring what pathway acupuncture or acupotomy decreases inflammatory factors(IL-1β and TNF-α). As mentioned earlier, PRRs in innate immunity include toll-like receptor (TLR) [68], which can activate transcription factors, such as NF-κB and MAPK, which in turn induce genes encoding enzymes and cytokines, leading to cartilage catabolism by upregulating IL-1β and TNF-α.Notably, TLR2/4 receptor expression was upregulated in KOA cartilage at the lesion site, but normal in normal cartilage without the lesion site [72]. The signal transduction pathways of TLR can be categorized into MyD88-dependent pathways and MyD88-independent pathways, with NF-κB is important in the downstream cascade of both pathways in TLR signaling [73]. It has been demonstrated that inhibition of PI3K/Akt can degrade IkBα and phosphorylate p65, which can block the transport of p65 from the cytoplasm to the nucleus and inhibit the NFκB signaling pathway [72]. Similar to NF-κB, MAPK can also regulate IL-1β and TNF-α, so inhibition of NF-κB or MAPK signaling pathways can provide new ideas for targeted therapy of KOA [95]. It has been suggested that electroacupuncture can inhibit DAMPs/TLR/NF-κB signaling pathway, reduce inflammatory response, and protect articular cartilage to achieve the purpose of treating KOA [74, 75].

### Macrophages mediate the production of MMP3 and MMP13 by IL-1β and TNF-α

**Fig. 7:**
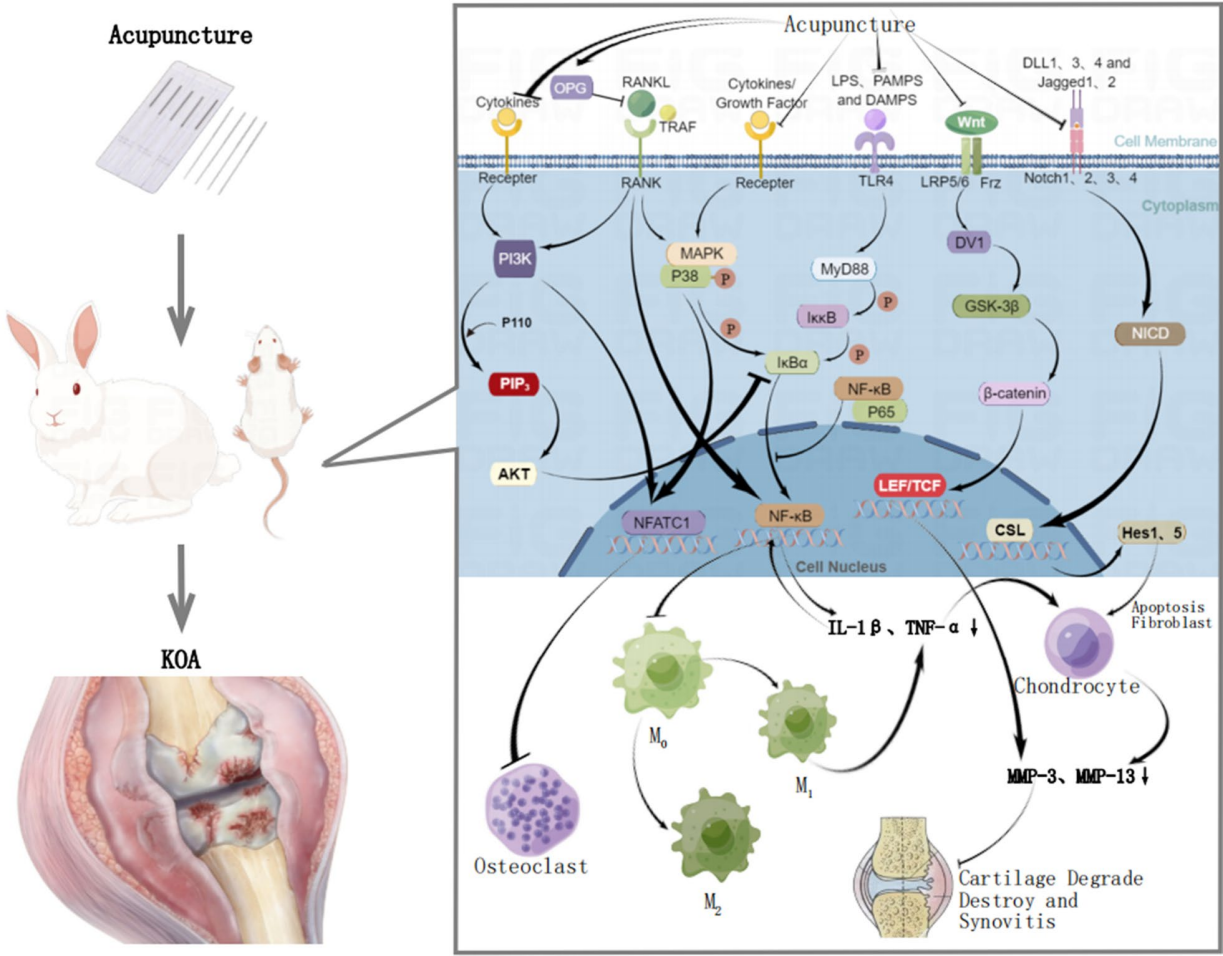
The main mechanism of acupuncture in animal models of KOA.The mechanism of KOA involves several inflammation pathways, NF-κB signaling is central to these inflammatory pathways. Acupuncture may have a therapeutic effect on KOA by inhibiting these pathways and reducing the destruction of cartilage and synovium by inflammatory factors and MMPs

Macrophages activated in the synovium produce inflammatory factors, which in turn stimulate chondrocytes to produce MMPs and aggregases [76]. It has been shown that depletion of macrophages can downregulate pro-inflammatory factors and prevent the formation of MMP-induced novel epitopes, thereby preventing cartilage destruction [77]. In KOA synovium, macrophages can regulate MMP production through a combination of IL-1β and TNF-α [78]. As well, in RA and KOA, the expression of MMP-3, and MMP-13 is dependent on NF-κB [79], and MMP3 and MMP13 are important in KOA synovium, and their expression is strongly up-regulated [80]. The results showed that acupuncture and Gao significantly decreased MMP-3 and MMP-13 levels in KOA animal models. Combined with the above studies, the mechanism may be that acupuncture inhibits macrophage polarization and down-regulates inflammatory factors such as IL-1β and TNF-α through NF-κB or MAPK. As a result, MMP3 and MMP13 are less likely to destroy and degrade synovial cartilage in KOA (Fig. 7).

### Other signaling pathways of KOA

Other signaling pathways closely related to KOA include: OPG/RANKL/RANK, Wnt/β-catenin, and Notch signaling pathway.

OPG/RANKL/RANK is an important immune-inflammatory pathway in KOA.RANK can bind to RANKL and then activates PI3K, MAPK, NF-κB and other signaling pathways [81]. OPG exerts its inhibitory effect on osteoclast differentiation and activity by preventing the binding of RANKL to RANK, thereby effectively inhibiting bone resorption [82]. It has been found that electroacupuncture can effectively reduce MMP13 and inhibit articular cartilage degeneration through the OPG/RANKL/RANK pathway [83].

Wnt/β-catenin: Wnt pathway is an important mechanism of cartilage development and bone remodeling [84]. Activation of β-catenin, by upstream Wnt family proteins can cause an increase in MMP3, MMP9 and MMP13, which can lead to cartilage ossification and degeneration [85]. Notably, electroacupuncture has been shown to downregulate MMP-13 expression and IL-1β production through the Wnt/β-catenin, consequently inhibiting cartilage matrix degradation, preventing chondrocyte apoptosis, and improving the morphology and structure of cartilage [31].

Notch signaling pathway: The mechanism of KOA is significantly associated with apoptosis of articular chondrocytes, and Notch is important in the regulation of chondrocyte proliferation, differentiation, and apoptosis [86, 87]. This signaling pathway exerts its influence on the differentiation and apoptosis of articular chondrocytes by activating downstream target genes, such as Hes1 and Hes5 [87] (Fig. 7).

### Clinical application

This study comprehensively analyzed the various factors of the efficacy of acupuncture-related therapies for KOA, including acupuncture methods, acupoints, and treatment duration, which can provide evidence-based basis for subsequent related animal experiments. So that they can be better applied to the clinic, and bring new therapeutic hope to KOA patients.

The results of this study showed that the commonly used acupoints for KOA were Dubi (ST35), Neixiyan (EX-LE4), Zusanli (ST36), Yanglingquan (GB34), Liangqiu (ST34), which are the acupoints around the knee joint and can be used clinically in the vicinity of the knee joint. In the study of different acupuncture methods, there was no significant difference in the course of treatment, so in the clinic, different acupuncture methods can be chosen according to the patient's willingness to accept them, and it is worth noting that acupuncture is effective for analgesia and inflammation in the early and middle stages of KOA, but there is no clear benefit in the late stages [7]. Our study showed that the duration of acupuncture treatment in most of the experiments was 15–20 min for 2–4 weeks, suggesting that in the clinic, the duration of acupuncture treatment can be controlled in this interval. Animal experiments are closely related to clinical trials, and it is hoped that higher quality articles will be published in the future to validate this idea, standardize acupuncture treatment, and provide clinicians with more reliable treatment options.

## Limitations

(1) The number of high-quality studies in the included studies was limited, and the investigators did not clearly describe the methods of randomization, allocation concealment and statistical blinding, resulting in selection bias and outcome measurement bias. (2) The heterogeneity of Meta results may reduce the level of evidence in the study conclusions, and subgroup meta-analysis and sensitivity analysis fail to reduce the heterogeneity, which may bias the results to some extent. (3) There are differences in different acupuncture points and treatment processes included in the study, which may increase clinical heterogeneity.

## Conclusions

Acupuncture-related therapy can be a possible treatment for KOA. The mechanism involves many immune-inflammatory pathways, which may be mediated by DAMPs/TLR/NF-κB/MAPK,PI3K/Akt/NF-κB pathway, or IFN-γ/JAK-STAT pathway. It needs to be further confirmed by more high-quality animal experiments or meta-analysis.

### Supplementary Information

**Additional file 1**. The PRISMA Checklist.

**Additional file 2**. Supplementary Figures.

## Data Availability

All data analysed during this study are included in this article and supplementary files.

## Abbreviations

KOA: Knee Osteoarthritis
MMP3: Matrix metalloproteinase-3
MMP13: Matrix metalloproteinase-13
SMD: Standardized mean difference
CI: Confidence interval
SUCRA: Surface under the cumulative probability curve area
Gn: Normal group
Gso: Sham operation group
Gm: Model group
Ga: Acupuncture group
Gao: Acupotomy group
Gmd: Medicine group
PAMPs: Pathogen-associated molecular patterns
DAMPs: Injury-related molecular patterns
LPS: Lipopolysaccharide
TRL: Toll-like receptor
