# Supplementary material for: Efficacy and immune-inflammatory mechanism of acupuncture-related therapy in animal models of knee osteoarthritis: a preclinical systematic review and network meta-analysis
Source: J Orthop Surg Res. 2024 Mar 8;19:177. doi: 10.1186/s13018-024-04660-9 (PMC10924386; doi:10.1186/s13018-024-04660-9)
Supplement: Supplementary file 2 — Additional file 2. Supplementary Figures. [file 13018_2024_4660_MOESM2_ESM.pdf]

	Sequence generation (selection bias)	Baseline characteristics (selection bias)	Allocation concealment (selection bias)	Random housing (performance bias)	Blinding (performance bias)	Random outcome assessment (detection bias)	Blinding (detection bias)	Incomplete outcome data (attrition bias)	Selective outcome reporting (reporting bias)	Other sources of bias (other)
Bao et al. 2011	+	+	?	?	?	?	?	+	+	+
Chen et al. 2020	+	+	?	?	?	?	?	+	+	+
Chen et al. 2022	?	+	?	+	?	+	?	+	+	+
Ding et al. 2017	+	+	?	+	?	?	?	+	+	−
Fu et al.2017	?	+	?	+	?	?	?	+	+	+
Fu et al. 2017	?	+	?	+	?	+	?	+	+	+
Gao et al. 2019	+	+	?	?	?	?	?	+	+	+
Hei et al. 2021	+	+	?	+	?	?	?	+	+	+
Huang et al. 2007	?	+	?	?	?	?	?	+	+	+
Huang et al. 2020	?	+	?	+	?	+	?	+	+	−
Jian et al. 2011	?	+	?	?	?	?	?	+	+	+
Ji et al. 2009	+	+	?	?	?	?	?	+	+	+
Liang et al. 2015	?	+	?	+	?	?	?	+	+	+
Liao et al. 2016	+	+	?	+	?	?	+	+	+	+
Li et al. 2011	?	+	?	+	?	?	?	+	+	+
Li et al. 2013	?	+	?	?	?	?	?	+	+	+
Li et al. 2016	?	+	?	?	?	?	?	+	+	+
Li et al. 2020	+	+	?	+	?	?	?	+	+	−
Lin et al. 2019	+	+	?	+	?	?	?	+	+	+
Liu et al. 2008	+	+	?	+	?	?	?	+	+	+
Liu et al. 2016	+	+	?	?	?	?	?	+	+	+
Liu et al. 2019	?	+	?	?	?	?	?	+	+	−
Liu et al. 2020	?	+	?	?	?	?	?	+	+	+
Liu et al.2021	?	+	?	+	?	+	?	+	+	−
Liu et al. 2021	+	+	?	+	?	?	?	+	+	−
Liu et al. 2021	?	+	?	+	?	+	+	+	+	−
Peng et al. 2020	+	+	?	+	?	?	?	+	+	+
Qin et al. 2022	?	+	?	+	?	?	?	+	+	+
Ruan et al. 2021	?	+	?	+	?	?	?	+	+	+
Shi et al. 2019	?	+	?	+	?	?	?	+	+	+
Sun et al. 2022	+	+	?	+	?	+	?	+	+	−
Tan et al. 2009	+	+	?	?	?	?	?	+	+	+
Tan et al. 2022	+	+	?	+	?	+	?	+	+	+
Wan et al. 2021	?	+	?	?	?	?	?	+	+	+
Wang et al. 2011	+	+	?	?	?	?	?	+	+	+
Wang et al. 2021	?	+	?	+	?	?	+	+	+	+
Wang et al. 2023	+	+	?	+	?	?	?	+	+	+
Wu et al. 2011	?	+	?	?	?	?	?	+	+	+
Wu et al. 2019	+	+	?	+	?	?	?	+	+	+
Wu et al. 2021	?	+	?	+	?	?	?	+	+	+
Wu et al. 2022	?	+	?	+	?	?	?	+	+	−
Xi et al.2016	?	+	?	+	?	?	?	+	+	+
Xi et al. 2016	?	+	?	?	?	?	?	+	+	+
Yang et al. 2019	?	+	?	+	?	+	?	+	+	−
Yuan et al. 2018	?	+	?	+	?	+	+	+	+	−
Yue et al. 2016	+	+	?	+	?	?	?	+	+	+
Yu et al. 2022	+	+	?	+	?	?	?	+	+	+
Zhang et al.2019	?	+	?	+	?	?	?	+	+	+
Zhang et al. 2019	+	+	?	+	?	?	+	+	+	+
Zhang et al. 2023	?	+	?	+	?	?	?	+	+	+
Zhang et al. 2023	?	+	?	+	?	?	?	+	+	+
Zheng et al. 2020	+	+	?	+	?	?	?	+	+	+
Zheng et al. 2023	+	+	?	+	?	?	?	+	+	+
Zhou et al. 2012	?	+	?	+	?	?	?	+	+	+
Zhou et al. 2023	+	+	?	+	?	?	?	+	+	+
Zhu et al. 2020	+	+	?	+	?	?	?	+	+	+

Supplementary Figure 1 Risk of bias

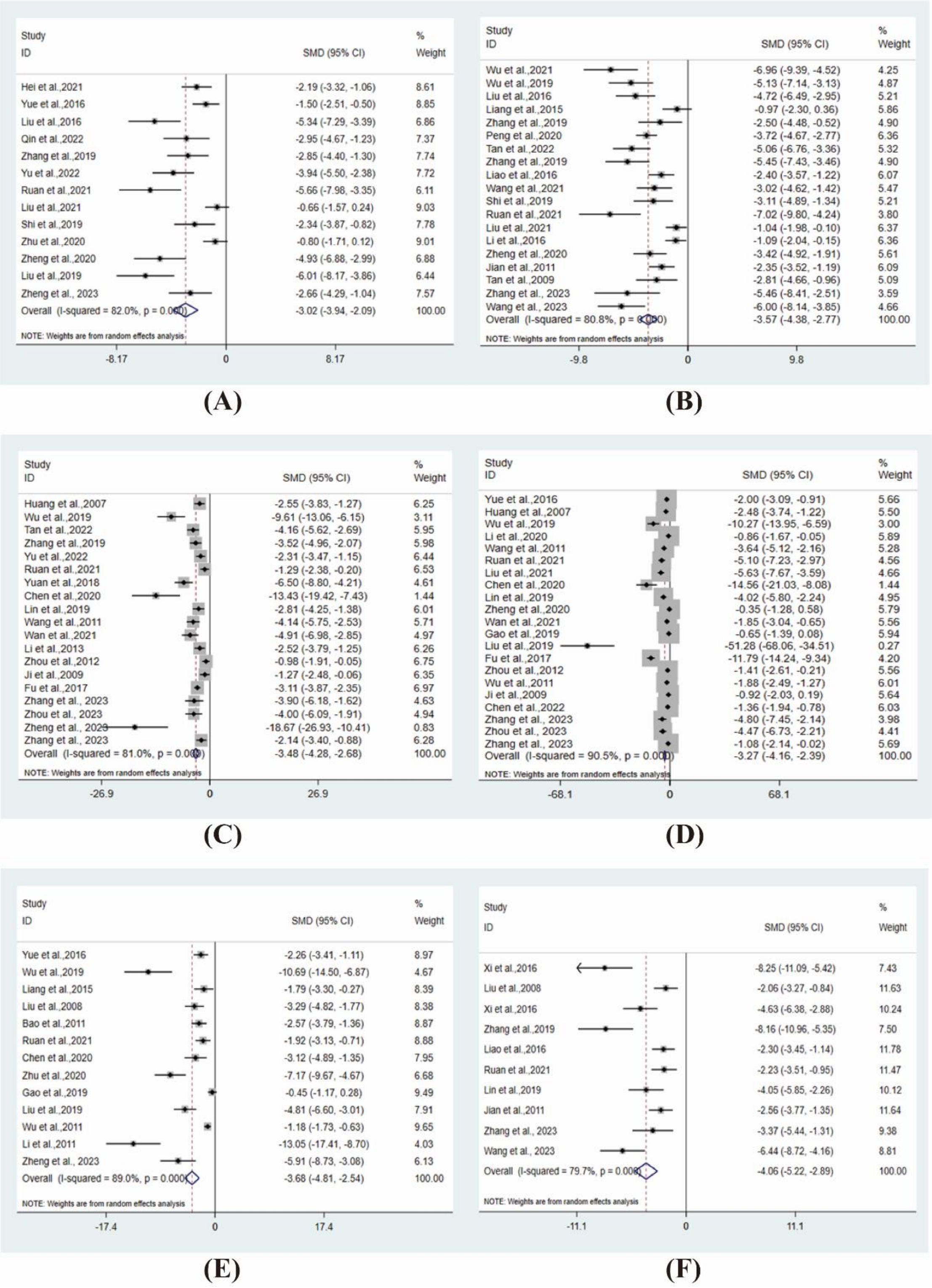

Supplementary Figure 2 Pair-wise meta-analysis

(A) Lequesne index scale; (B) Mankin score; (C) IL-1β; (D) TNF-α; (E) MMP-3; (F)MMP-13

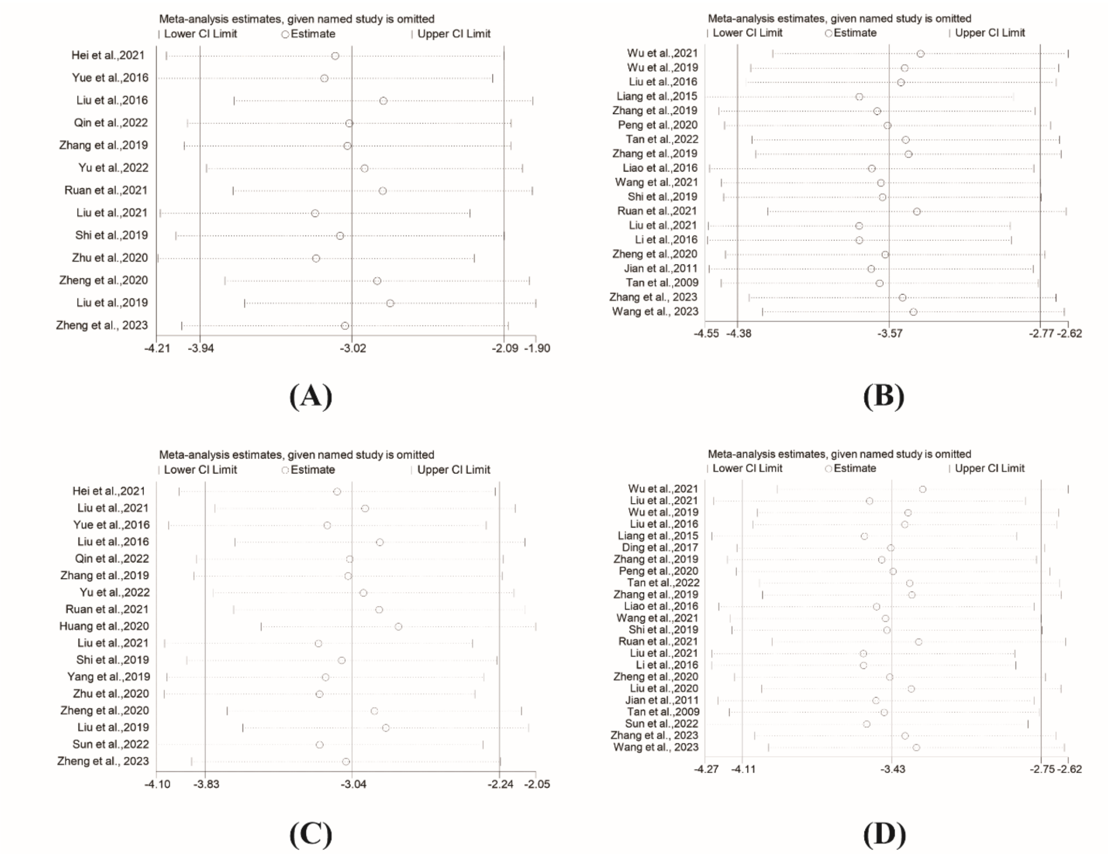

Supplementary Figure 3 Sensitivity analysis

(A) acupuncture methods of Lequesne index scale; (B) acupuncture methods of Mankin score; (C)treatment courses of Lequesne index scale; (D)treatment courses of Mankin score

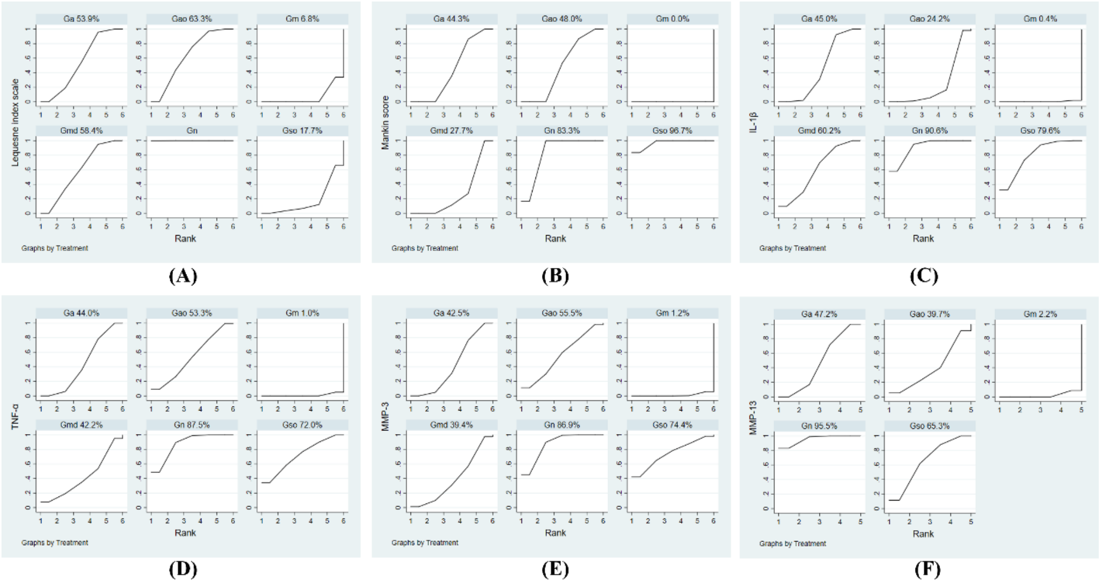

Supplementary Figure 4 SUCRA results

(A) Lequesne index scale; (B) Mankin score; (C) IL-1β; (D) TNF-α; (E) MMP-3; (F)MMP-13
